# Tumor-infiltrating lymphocyte: features and prognosis of lymphocytes infiltration on colorectal cancer

**DOI:** 10.1080/21655979.2022.2162660

**Published:** 2023-01-12

**Authors:** Miao Qin, Gang Chen, Jinxia Hou, Li Wang, Qunfeng Wang, Lina Wang, Dan Jiang, Ye Hu, Bei Xie, Jing Chen, Hulai Wei, Guangxian Xu

**Affiliations:** a Key Laboratory of Preclinical Study for New Drugs of Gansu Province, School of Basic Medical Sciences, Lanzhou University, Lanzhou, China; b Guangdong Provincial Key Laboratory of Medical Molecular Diagnostics, School of Medical Technology, Institute of Clinical Laboratory, Guangdong Medical University, Dongguan, China; c Gastroenterology, Xinhua Hospital, Shanghai Jiao Tong University School of Medicine, Shanghai, China

**Keywords:** CRC, infiltrating features, CD8+TILs, prognosis value, ICB response

## Abstract

Tumor-infiltrating lymphocytes (TILs) are vital elements of the tumor microenvironment (TME), and the anti-tumor activity of TILs on colorectal cancer (CRC) has been a topic of concern. However, the characteristics and prognosis of the various types of lymphocyte infiltration in CRC have not been fully explained. Our study aimed to identify distinct features and prognosis of TILs. We integrated multiple-cohort databases to illustrate the features, proportions, and prognosis of TILs on CRC. We found that macrophages were significantly enriched in CRC. When we used the scRNA-seq database to further evaluate the proportion of TILs, we noticed markedly higher numbers of CD4 + T cell, B cell, and CD8 + T cell in four Gene Expression Omnibus Series (GSE) CRC cohorts. Interestingly, we found that the infiltrating level of TIL subgroups from highest to lowest is always dendritic cells, CD8 + T cells, CD4 + T cells, neutrophils, B cells, and macrophages; the proportion of infiltration is largely constant regardless of mutations in specific genes or somatic copy number variation (sCNV). In addition, the data corroborated that CD4+ TILs and CD8+ TILs have certain application values in the prognosis of CRCs, and age negatively related to CD8+ TILs and B plasma infiltration. Finally, patients with CRC who are older than 70 years have a better response to immune-checkpoint blockade.

## Graphic Abstract

**Figure:**
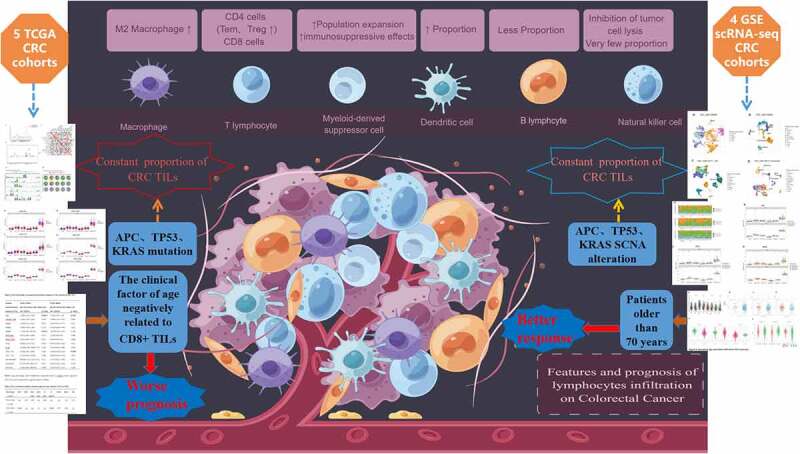

## Introduction

Colorectal cancer (CRC) is the third most common cancer in the world, with 1.8 million diagnosed cases in 2018. It is the second leading cause of cancer death globally (883,200 cases), only second to lung cancer (1,766,400 cases) [1,2]. In China, CRC is still a heavy public health burden; the standardized mortality rates of CRC experienced a significant upward trend for both sexes, which ranged from 4.7 to 5.8 per 100,000 in a decade [3]. Most patients with early CRC were cured by surgery, or surgery combined with radiotherapy and chemotherapy. However, the treatment of patients with mid-advanced CRC remains a challenging clinical research focus. How to completely eradicate CRC and prevent metastasis and postoperative recurrence remains a crucial clinical issue that requires urgent remedy.

In the last decade, cancer immunotherapy has made striking progress in the management of advanced and metastatic tumors. The remarkable achievements of adoptive cell therapies (ACTs) using tumor-infiltrating lymphocytes (TILs) have drawn substantial focus. ACT using naturally occurring tumor-reactive lymphocytes can mediate complete regression in patients with melanoma [4]. TIL infusion in combination with chemotherapy or radiotherapy achieved a 40–72% clinical response rate, with nearly 40% of patients with melanoma having complete response (CR) and remaining relapse-free for more than 7 years [5]. Galon et al. [6] in 2006 for the first time described that different subgroups of TIL could predict the prognosis of CRC. Subsequently, more studies proved that TILs are closely related to the prognosis of CRC and tumor regression degree after neoadjuvant radiotherapy for advanced rectal cancer [7]. The cytotoxicity of CD8 + T cells plays an important role in the mechanism of anti-tumor immunity. A large number of studies have shown that CD8 + T cells are associated with the benign prognosis of CRC [6–9], esophageal cancer [10], ovarian cancer [11], renal cell carcinoma [12], and pancreatic cancer [13]. Ling et al. [14] found that the infiltration of CD8 + T cells in CRC tumor epithelium provided strong prognostic information. On the contrary, the immune suppressive cells, such as M2 macrophages and myeloid-derived suppressor cells reach their highest infiltrating expression level in CRC, but reveal poor prognosis [15,16]. Tumor cell recognition by CD8 + T cells is prevented by regulatory T cells (Treg), myeloid-derived suppressor cells (MDSC), tumor-associated (M2) macrophages, and immune checkpoint regulators expressed by tumor cells [17]

On these bases, immune cells infiltrating or encircling colorectal primary tumors, particularly TILs, represent an important prognostic factor [18,19]. TILs have been shown to have predictive and prognostic value in a variety of solid cancers, including melanoma [20,21], lung cancer [22,23], and CRC [24,25]. Different subsets of infiltration lymphocytes in the primary tumor may predict the differing outcomes of patients with CRC. Although several studies have indicated that some checkpoint genes are related to the TIL load and the overall survival of patients with CRC and pan-cancer [26–29], few studies have analyzed the TILs infiltration features using single-cell RNA sequencing (scRNA-Seq) in the Cancer Genome Atlas (TCGA) cohorts. Also, the relationships between some cancer suppressor genes (such as Tumor Protein P53 (TP53, P53), APC Regulator of WNT Signaling Pathway (APC), and Phosphatidylinositol-4,5-Bisphosphate 3-Kinase Catalytic Subunit Alpha (PIK3CA)) mutation and prognosis and TIL infiltration features have not been fully analyzed.

Based on the fact that the proportion of infiltration in different subgroups is closely related to the prognosis and treatment response of patients with CRC, we speculate whether different genetic mutations, somatic variants, and other states, as well as clinical factors, such as age, may influence the redistribution of TILs infiltration characteristics, thus, indirectly affecting the prognosis and immunotherapy response of patients with CRC. The study provided a novel approach to evaluating the features and prognosis of TILs on CRC using a variety of existing immuno-infiltration and scRNA-Seq databases. These were used to analyze the infiltration characteristics and prognosis of various types of TILs. We proposed a correlation between age and TILs in patients with CRC, as well as that between age and ICB responses, which may be an important reason for the relevance of age to the prognosis and treatment of CRC. This provides a valuable basis for the prognostic and immunotherapeutic assessment of patients with CRC.

### Materials and methods

#### TIMER 2.0

TIMER 2.0 [30–32] (available from http://timer.cistrome.org) provides four modules (including gene differential expression, outcome, mutation, and correlation) for investigating the associations between immune infiltrates and genetic or clinical features, and four modules for exploring cancer-related associations in TCGA cohorts. The lymphocytes infiltrating level data of five TCGA CRC samples were collected from TIMER 2.0 ‘Estimation’ module (see supplementary material for data details). To make immune infiltration estimations more convincing, TIMER 2.0 utilizes an R package, which integrates six different algorithms: Tumor IMmune Estimation Resource (TIMER) (available from https://cistrome.shinyapps.io/timer/ [33], CIBERSORT (available from https://cibersortx.stanford.edu/) [34], QUANTISEQ R package (quanTIseq) [35], cell types enrichment analysis (X-Cell) (available from http://xcell.ucsf.edu/)

[36], MCP-counter R package (MCP-counter) [37] and EPIC (available from https://gfellerlab.

shinyapps.io/EPIC_1-1/) [38]. Using these algorithms to uniformly pre-process all TCGA samples, users would reach more credible conclusions through estimation. To further investigate the immune infiltration distribution difference, we utilized TIMER 2.0 to evaluate the infiltration proportion SCNA and gene mutation conditions of specific genes.

### The Cancer Immune Single-Cell Hub (TISCH)

Our data were obtained from the CRC scRNA-seq provided by TISCH, and only four cohorts of patients with primary CRC who had no other treatment were included in this study. We excluded studies of patients who already had radiotherapy and those with metastatic colon cancer. TISCH [39] (http://tisch.comp-genomics.org/home) is a scRNA-seq database for investigating the tumor microenvironment (TME). TISCH can be used to explore in detail the various immune cell-types in different cancers of the TME at the single-cell level. We used TISCH to further analyze the infiltration proportion of CRC TILs at the single-cell level. CRC_GSE108989 [40] used Smart-seq2 platform to evaluate 11,125 cells from 12 patients with CRC. CRC_GSE139555 [41] used 10x Genomics platform to measure 10,112 cells from two patients with CRC. Similarly, CRC_GSE146771_10x and CRC_GSE146771_Smartseq2 are single-cell sequencing of 15,000 and 20,000 cells from 10 patients with CRC using two different methods from the same datasets [42]. A standardized analysis process was used by TISCH for each dataset to carry out quality assurance, clustering, and cell-type annotation.

### Xiantao academic

**Figure 1.**
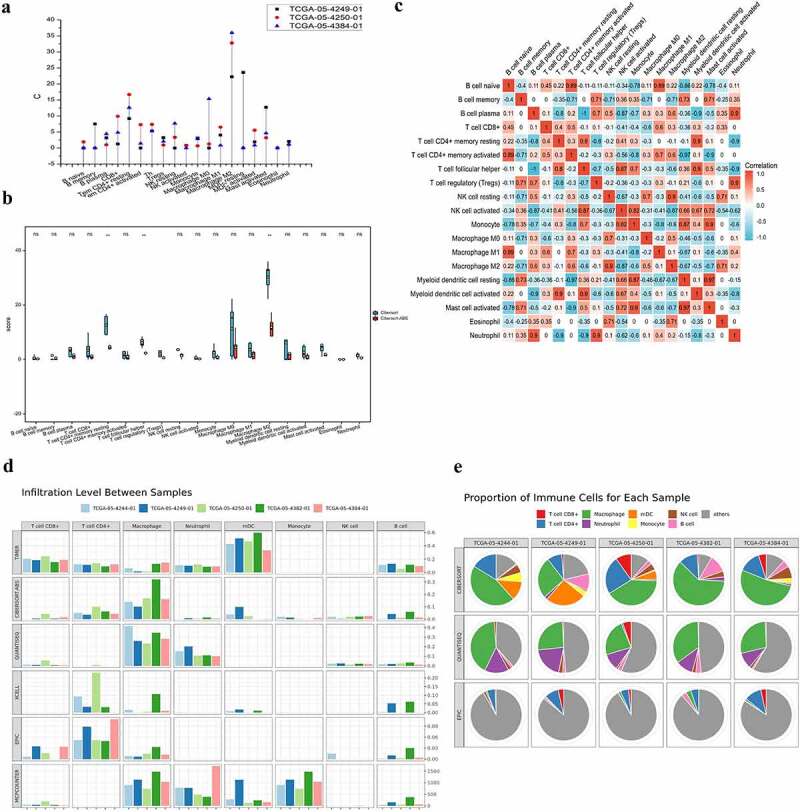
The infiltrating features and proportion of each lymphocyte in CRC TCGA tissue samples. **(A)** The infiltrating levels of each lymphocyte in three CRC TCGA cohorts by TIMER 2.0. The vertical figure was drawn using Origin Pro Portable software. **(B)** Infiltration of immune cells calculated by two algorithms (CIBERSORT and CIBERSORT-ABS) of CRC was presented using TIMER 2.0. CIBERSORT group is marked blue, and CIBERSORT-ABS is marked red. Open circles represent mean value. Data were analyzed using Wilcoxon rank sum test. **(C)** The correlation map of the proportion of each immune cell in CRC samples calculated using CIBERSORT algorithms provided by TIMER 2.0. Red and blue colors indicate positive and negative correlations, respectively. Both multiple-panel bar plot (B) and corrplot (C) are demonstrated using Xiantao Academic (www.xiantao.love). **(D-E)** For the eight infiltrating lymphocyte cell types for which all six algorithms can calculate the abundance, a multi-panel bar plot and component proportions pie chart drawn using TIMER 2.0 show differences of their infiltration level estimated by different algorithms among five different TCGA tissue samples. CRC, Colorectal cancer; TCGA, The Cancer Genome Atlas; TIMER, Tumor Immune Estimation Resource; NK, natural killer.

The multiple-panel bar plot (Figure 1b) and correlation plot (Figure 1c) are demonstrated using Xiantao Academic (https://www.xiantao.love). The online platform has internally installed statistical analysis and visualization software – R language (version 3.6.3), and the R package mainly used ggplot2 to visualization. The data were also collected from TIMER 2.0.

### ASSISTANT FOR Clinical bioinformatics

The expression of eight classical immune checkpoints and the responses of patients with CRC to immunotherapy were analyzed using ASSISTANT FOR Clinical Bioinformatics (www.acbl2.com). The TCGA dataset (available at https://portal.gdc.com) was used to download the RNA-sequencing expression profiles and associated clinical data for CRC. Using 50 years as the cutoff age, the sample size for patients with CRC younger and older than 50 years was 74 and 538, respectively. These eight genes’ expression values were extracted after they were chosen as immune-checkpoint-relevant transcripts: SIGLEC15, TIGIT, CD274, HAVCR2, PDCD1, CTLA4, LAG3, and PDCD1LG2.

As for the 712 patients with CRC enrolled in the study, the Tumor Immune Dysfunction and Exclusion (TIDE) algorithm [43,44] indicated a potential ICB response. The TIDE algorithm uses a set of gene expression markers to assess two different mechanisms of tumor immune escape, including dysfunction of tumor-infiltrating cytotoxic T lymphocytes (CTL) and rejection of CTL by immunosuppressive factors. The higher the TIDE score, the less effective the immune checkpoint blockade (ICB) therapy and the shorter the survival after receiving ICB. R Foundation for Statistical Computing (2020) version 4.0.3 implemented all the aforementioned analysis techniques and R package was used for the ggplot2 and heatmap. All data were analyzed and visualized by R v4.0.3 built into this online analysis platform.

### CRI iAtlas portal

We used the CRI iAtlas Portal (https://isb-cgc.shinyapps.io/iatlas/) [45] to analyze the impact of the lymphocyte infiltration level on patient survival, and whether a pattern also exists in different tumors where patients respond better to ICB treatment with increasing age. We analyzed three different tumor cohorts to examine the response of patients to ICB treatment using age as a variable. Cohort studies of Gide et al. [46], Hugo et al. [47], and Prins et al. [48] report the effectiveness of ICB in the treatment of melanoma and glioblastoma. We used age as variables to analyze the association between age and ICB response.

### Origin pro 8.5 and fig draw

We used Origin Pro 8.5 Software to draw the vertical drop-line figure (Figure 1a) to intuitively reflect the composition and infiltration proportions of the TILs of three CRC TCGA cohorts. The data were also collected from TIMER 2.0. The graphical abstract at the beginning of this article was drawn by Fig Draw (https://www.figdraw.com/static/index.html#/).

### Statistical analysis

All statistical analyses were performed in different versions of R that were installed in TIMER 2.0, TISCH, Xiantao Academic, CRI iAtlas Portal, and ASSISTANT FOR Clinical Bioinformatics. TCGA Cox hazard regression analysis or Kaplan–Meier method was used to evaluate the prognostic value of TILs’ clinical characteristics and their subgroups. The Cox regression findings, including hazard ratios (HRs) and statistical significance, are automatically produced by TIMER 2.0. A statistically significant P value was set at 0.05.

### Results

This study was conducted to investigate the distribution, infiltration characteristics, and prognostic impact of TILs in CRC. We hypothesized whether the distribution of each subpopulation of TILs differs under different gene mutations and somatic copy number variants; thus, indirectly affecting the prognosis of patients with CRC and their responses to immune checkpoint inhibitor. It was found that the TIL distribution profile remained constant across mutations and somatic copy number variants of specific genes. The level of infiltration of subpopulations of TILs from highest to lowest was always DC cells, CD8 + T cells, CD4 + T cells, neutrophils, B cells, and macrophages. Surprisingly, however, we found a negative correlation between age and CD8+ TILs and a better response to immune checkpoint therapy in patients with CRC older than 70 years, suggesting that age may be a very important factor in patient prognosis and immunotherapy.

### Preliminary exploration of the characteristics and proportion of lymphocyte infiltration in CRC

In order to estimate the infiltrating features of TILs, we used TIMER 2.0, TISCH to investigate the associations between immune infiltrates and genetic or clinical features in the TCGA cohorts. As shown in Figure 1, the immune suppressive cells, the M2 macrophages, reach their highest infiltrating expression level in three TCGA cohort of CRC, followed by myeloid dendritic cell (mDCs) resting, Tem CD4+ resting, CD8+, and M0 macrophages (Figure 1a).

Furthermore, we explored the infiltrating level of each immune cell in CRC tissue using two algorithms (CIBERSORT and CIBERSORT-ABS). Consistent with the above result, the highest infiltrating cells were M2 macrophages, M1 macrophages of antitumor cell function are the opposite. This was followed by the Tem CD4+ resting and T cell follicular helper (p < 0.01). It has been demonstrated that Tregs and mDCs are enriched in CRC; the difference, however, was not statistically significant (Figure 1b).

Correlation maps were used to assess the correlation among each lymphocyte. The closer the value to 1, the greater the correlation becomes. Among the B cell naïve and T cell CD4+ activated (0.89), B cell naive and Macrophage M1 (0.89), B cell plasma and Neutrophil (0.9), T cell CD4+ memory resting and mDC resting (0.9), T cell follicular helper (Th) and monocyte (0.87), Th and mDC activated (0.9), Tregs and Neutrophil (0.9), the correlation coefficient was larger than 0.85 (p < 0.01). In contrast, negative correlations were observed among B cell naïve and mDCs resting (−0.86), B cell plasma and monocyte (−0.87), B cell plasma and mDC resting (−0.9), T cell CD4+ memory resting and Neutrophil (−0.9), T cell CD4+ memory activated and mDC resting (−0.97), T cell CD4+ memory activated, and Mast cell activated (−0.9) (Figure 1c, Supplementary Figure 1).

Taking tissue specificity and difference of algorithms into account, we estimated six types of immune cell populations. The results obtained from TIMER 2.0 of five TCGA CRC samples are shown in Figure 1d and e. Macrophages were significantly enriched in CRC. In addition, a high proportion of CD4+, CD8+, mDC, monocytes, and others was observed.

### The signature and proportion of lymphocyte infiltration in CRC of single-cell RNA sequence (scRNA-seq)

**Figure 2.**
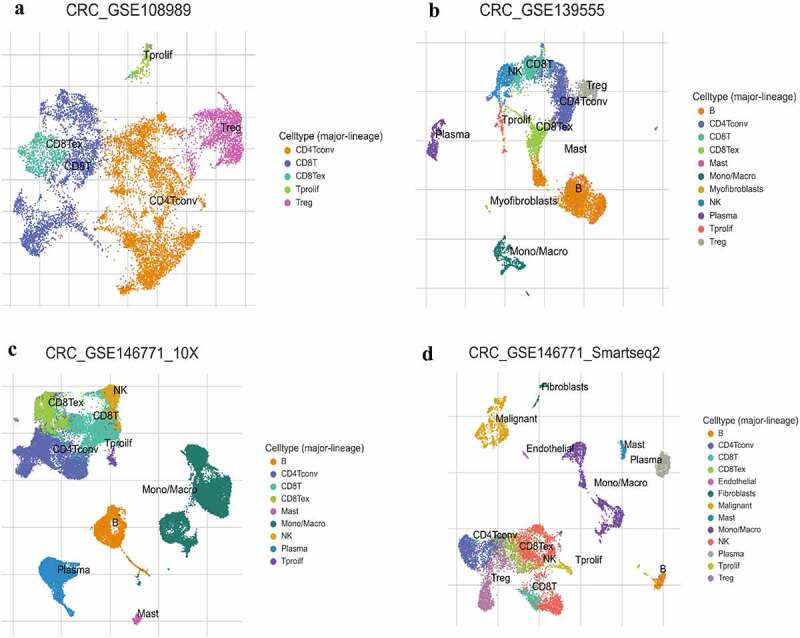
The infiltration level of major-lineage immune cells in CRC GSE sc-seq samples. In single cell level, four different GSE sc-seq samples of primary CRC tissues were analyzed to evaluate the proportion of each cell type used TISCH (a-d). GSE, Gene Expression Omnibus Series; CRC, Colorectal cancer; TISCH, The Cancer Immune Single-Cell Hub.

To further investigate the proportion of the infiltrating lymphocyte cells in single-cell level, we analyzed the infiltrating level of each major lineage (cell type) of four CRC GSE scRNA-seq samples using TISCH. What stands out is the markedly higher rates of CD4 + T conventional cell, B cell, and CD8 + T cell (Figure 2a-b, d); only one GSE scRNA-seq sample showed a higher proportion of monocyte/macrophage infiltration (Figure 2c). In view of tumor heterogeneity, the single-cell sequence may reflect the results more precisely.

### The distribution of infiltration lymphocyte cell on the condition of frequent gene mutation and SCNA alteration

**Figure 3.**
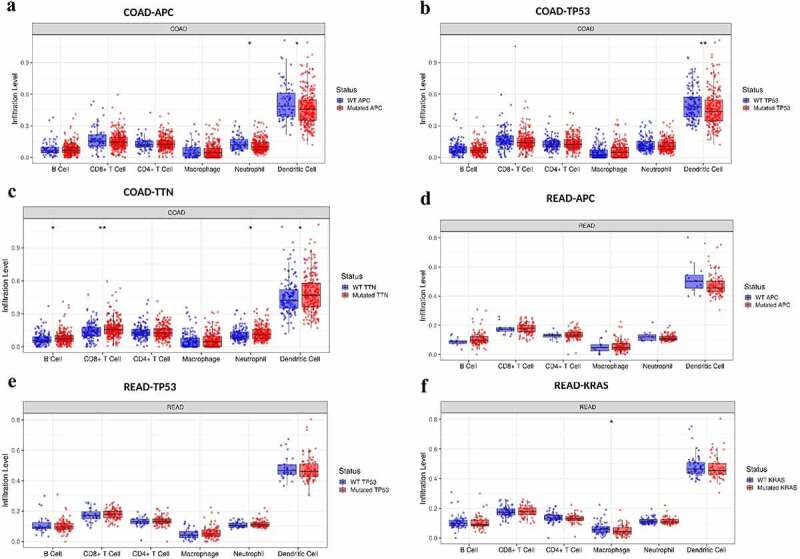
The distributions of immune infiltration levels under different gene mutation status.

The next section of the study was concerned with comparing the distributions of immune infiltration levels under different gene mutation status. The result as shown in Figure 3 reveals that the three genes arranged according to mutation frequency were APC Regulator of WNT Signaling Pathway (APC) (80.7%), Tumor Protein P53 (TP53) (63.5%), and Tenascin N (TTN) (63.5%) in CRC tissues (Figure 3a-3c). The lymphocyte infiltration level in wild-type APC was higher than that of mutant APC, and the infiltration level of DCs was the highest, followed by neutrophils (P < 0.05) (Figure 3a). Both the infiltrating DC and CD8 + T cell levels were higher in wild-type TP53 cells than in the mutant type (Figure 3b). In addition, the lymphocyte infiltration level of wild-type TTN gene was lower than that of mutant TTN, and the infiltration level of immune cells from highest to lowest was: DC cells (P < 0.05), CD8 + T cells (P < 0.01), CD4 + T cells (P > 0.05), neutrophils (P < 0.05), B cells (P < 0.05), and macrophages (P > 0.05) (Figure 3c).

**A-C**: The top three genes with the most frequent non-synonymous mutations of colon cancer (COAD) tissues evaluated using TIMER 2.0. **D-F**: The top three genes with the most frequent mutations of rectal cancer (READ). Statistical significance was estimated using a two-sided Wilcoxon rank-sum test. *P*-value Significant Codes: *** means *p* < 0.001, ** means *p* < 0.01, * means *p* < 0.05. APC: APC Regulator of WNT Signaling Pathway; TP53: Tumor Protein P53; TNN: Tenascin N; PIK3CA: Phosphatidylinositol-4,5-Bisphosphate 3-Kinase Catalytic Subunit Alpha; KRAS: Oncogene KRAS2.

Consistent with colon adenocarcinoma (COAD) result, APC (88.5%), TP53 (74.6%), KRAS (61.6%), and TTN (45.9%) were the highest mutation frequency genes in rectal adenocarcinoma (READ). The distributions of lymphocyte infiltration level of wild-type APC/TP53 was higher than that of mutant APC/TP53 (P > 0.05) but had no statistical difference, and the infiltration level of DC was the highest, followed by CD8 + T cell, CD4 + T cell, and others (Figure 3d-e). The level of macrophage infiltration of wild-type Oncogene KRAS2 (KRAS) gene was higher than that of mutant KRAS (P < 0.05) in READ. Based on the above results, from high-infiltration to low-infiltration, the distribution of lymphocyte cells were DC cells, CD8 + T cells, CD4 + T cells, neutrophils, B cells, and macrophages. The highest mutation frequency genes were APC, TP53, TTN, KRAS, and PIK3CA (data not shown) in CRC.

**Figure 4.**
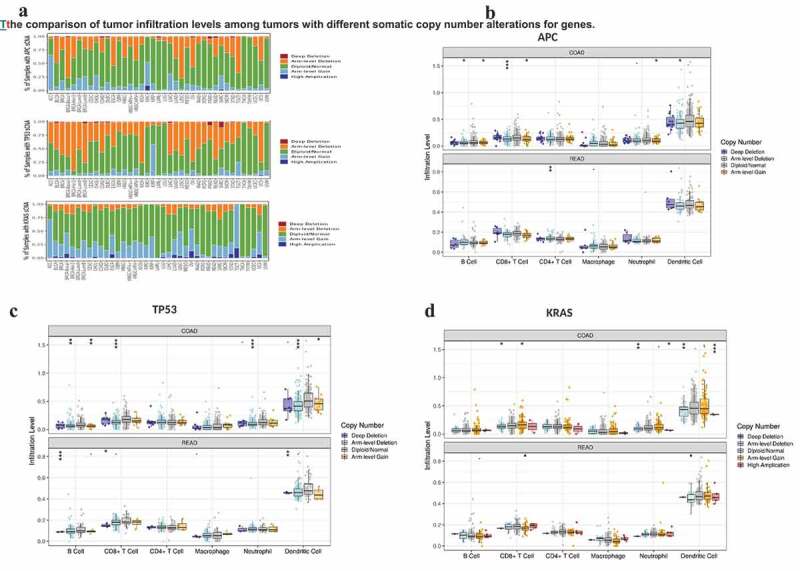
The comparison of tumor infiltration levels among tumors with different somatic copy number alterations for genes.

Further analysis showed that the distribution of TILs of different types of somatic copy number variation (SCNA) occurred in specific genes of CRC (Figure 4). Figure 4a showed the distributions of each immune subset at each copy number status in selected cancer types. In COAD and READ, TP53 arm-level deletion was most likely to occur; however, APC and KRAS chromosomes often existed as diploid/normal. Arm-level deletion and gain sporadically occur.

The distribution of immune cells was similar to that in Figure 3, the infiltrating levels of immune cells from high to low were DC cells (APC chromosome was mainly absent in the whole arm, P < 0.05), CD8 + T cells (APC chromosome had whole-arm deletion or arm-level increase, P < 0.05), CD4 + T cells, neutrophils, B cells (whole-arm loss and increase, P < 0.05), and macrophages (Figure 4b). The features of infiltrating immune cells are similar to the characteristics of the infiltrating proportion of APC under the condition of chromosome with SCNA alteration. The distribution of DC in somatic cells with various copy number variants was highest, consisting largely of whole arm-level increase and deletion (P < 0.0001), followed by CD8 + T cells (TP53 chromosome had whole-arm loss, P < 0.0001), CD4 + T cells, neutrophil (arm-level deletion), P < 0.0001), B cells (arm-level deletion, P < 0.01), and macrophages (Figure 4b). SCNA occurred in KRAS with similar levels of infiltration of immune cells as explained above, and SCNA was mainly about arm-level gains and arm-level deletions or gain, and high-amplification (Figure 4d). What stands out of above results is the consistent trend that the infiltration level of TILs in CRC from highest to lowest were DC cells, CD8 + T cells, CD4 + T cells, neutrophils, B cells, and macrophages, regardless of the specific genes point mutated or the occurrence of SCNA.

### The clinical characteristics and prognosis shown in each subset of CRC TILs

**Figure 5.**
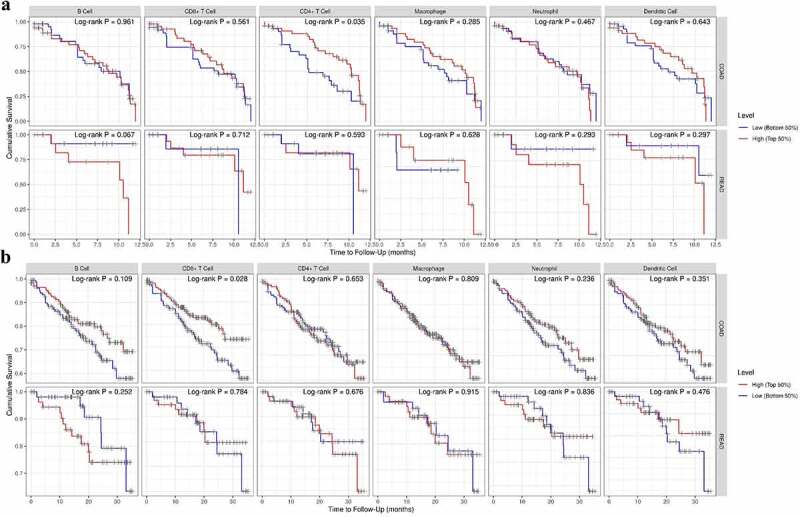
The survival difference of CRC TILs of each subset. To visualize the survival differences measured by TIMER 2.0, the Kaplan–Meier plots for immune infiltrates of CRC are shown in A-B. A: 12-month follow-up; B: 36-month follow-up. The median divides the infiltrating levels of each subset into two categories: low and high. each graphic displays the P-value of a log-rank test used to compare two groups’ survival curves.

In the final part of the study, the Cox regression and survival difference of each subset of TILs were assessed using TIMER 2.0. We investigated the clinical significance of tumor immune subgroups in CRC by correcting for various factors in a multivariable Cox proportional hazard model. Several clinical characteristics, such as age, gender, ethnicity, and tumor stage, were used as covariates. We found that age [HR = 1.036 (1.012–1.060), P = 0.003] and TNM stage 4 [HR = 7.718 (2.282–26.109), P = 0.001] were independent prognostic factors in COAD. Similarly, age [HR = 1.135 (1.031–1.25), P = 0.010] and CD8 + T cell [HR = 0.009 (0.000–1.896), P = 0.049] were independent prognostic factors in READ (Table 1). The results obtained from the preliminary analysis of survival difference of CRC TILs are set out in Figure 5(a-b). Higher infiltrating level of CD4+ TILs reveal a better prognosis in COAD at 12-month follow-up (p = 0.035, Figure 5a). Not surprisingly, higher level of CD8+ TILs demonstrated better prognosis in COAD at 36-month follow-up (p = 0.028, Figure 5b). The reason why we have not seen similar results in READ may be explained by the fact that the number of patients with rectal cancer was small (N = 80, Table 1).

CRC, colorectal cancer and TIL, tumor-infiltrating lymphocytes

**Table 1.** The multivariate Cox proportional hazard analyses of the subsets of CRC TILs.

Clinical characteristics/ subsets of TILs	TCGA-COAD [N = 253,n(live) = 190, n(die) = 63]		TCGA-READ [N = 80, n(live) = 66, n(die) = 14]	
	HR (95%CI)	p-value	HR (95%CI)	p-value
Age	1.036(1.012–1.060)	0.003**	1.135(1.031–1.25)	0.010*
Gender_male	1.245(0.726–2.132)	0.426	1.494(0.216–1.031)	0.684
Stage2	1.318(0.412–4.214)	0.641	3.930(0.044–3.477)	0.401
Stage3	2.564(0.793–8.294)	0.116	6.220(0.089–4.348)	0.632
Stage4	7.718(2.282–26.109)	0.001**	1.970(0.018–2.187)	0.186
Race_Black	0.541(0.064–4.569)	0.573	2.148(0.000-Inf)	0.998
Race_White	0.501(0.062–4.044)	0.517	4.834(0.000-Inf)	0.999
Purity	0.685(0.145–3.231)	0.633	9.971(0.028–3.494)	0.442
B_cell	32.559(0.042–25,494.775)	0.306	5.240(0.000–9.298)	0.940
CD8_T cell	0.009(0.000–1.896)	0.084	0.000(0.000–8.210)	0.049*
CD4_T cell	0.079(0.000–24.608)	0.386	0.000(0.000–4.523)	0.457
Macrophage	39.692(0.110–14,378.733)	0.221	4.772(0.000–7.769)	0.164
Neutrophil	0.073(0.000–3884.177)	0.637	9.678(0.000–4.223)	0.320
Dendritic	3.193(0.038–269.042)	0.608	4.320(0.015–1.214)	0.138

Age and Stage 4 were independent prognostic factors in COAD, similarly, age and CD8_T cell were independent prognostic factors in READ.

In addition, we also calculated the relationship between immune cell infiltration and clinical parameters such as age, gender, race, tumor purity, clinical stage, and grade. There was no significant correlation between the subsets of each lymphocyte and gender, race, tumor purity, tumor stage, or grade (P > 0.05) using stratified analysis and Cox regression. CD4 + T cells, naive CD4 + T cells, B memory cells, and NK cells were all positively linked with age in colon cancer tissue samples (TCGA-COAD). The infiltration of CD8 + T cells and plasma B cells were negatively correlated with age in rectal cancer tissue samples (TCGA-READ), but the infiltration of memory CD4 + T cells, monocytes, and macrophages were positively correlated with age, as shown in Table 2. We conducted a correlation analysis to further explore whether age as an important clinical factor is vital for influencing TILs infiltration patterns in CRC tissues. We found that CD8+ TILs and B plasma are negatively related with age. According to our research, age, CD8+ TILs, and CD4+ TILs all play a vital role in the prognosis of CRC. Thus, the immune cell infiltration patterns cannot be utilized to determine the clinical stage and tumor grade of CRC and age may be one of the most important factors influencing TIL infiltration patterns in CRC tissues. With advancing age, the infiltration features of each subgroup of TILs may transform as the tumor progresses.

**Table 2.** The correlation analysis between age and each subsets of TILs of CRC.

Tissue Type Z-score	CD8+	CD4+	CD4+ naive	CD4+ mem	B mem	B plasma	Mono	Macro	NK
TCGA-COAD (N = 458)	n.s	2.432	2.904	n.s	2.649	n.s	n.s	n.s	2.122
TCGA-READ (N = 166)	−2.212*	n.s	n.s	2.5	n.s	−3.133*	2.157	2.651	n.s

**Z-score: increased risk (Z > 0, P < 0.05)

Z-score: decreased risk (Z < 0, P < 0.05)

n.s: not significant (P > 0.05)

We found that CD8+ TILs and B plasma are negatively related to age. With age changing, the infiltration features of subgroups of TILs also change consequently. CD4+ mem:CD4+ memory; B mem: B memory; Mono: Monocyte; Macro: Macrophage.

### Patients with CRC older than 70 years respond better to ICB

**Figure 6.**
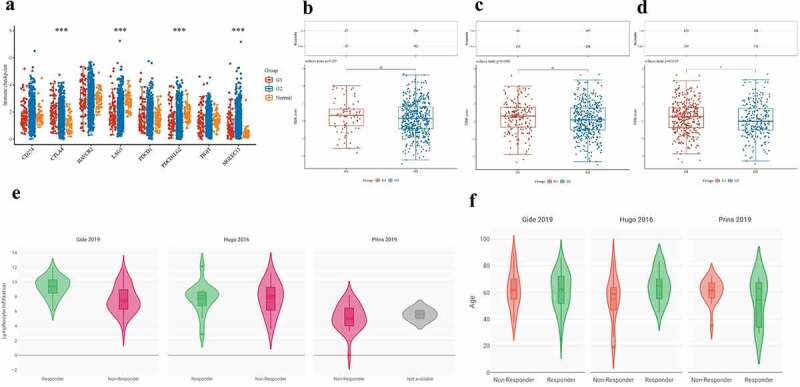
Increasing age associated with better ICB responses. A: The distribution of various immune checkpoint genes’ expression in CRC tissues and healthy tissues was analyzed using ASSISTANT FOR clinical bioinformatics. different colors denote different groups of samples in the abscissa, while the ordinate shows the distribution of gene expression. Asterisks (*) indicate significance levels. *p < 0.05, **p < 0.01,***p < 0.001. The statistical difference of the two groups was compared using the Wilcox test. **B-D**: The association between different age groups and ICB responses was analyzed using ASSISTANT FOR Clinical Bioinformatics. The top graph shows the table of CRC sample immune response statistics among different groups in the prediction results, and the bottom graph shows the distribution of immune response scores among different groups in the prediction results. The TIDE algorithm was used to forecast potential ICB responses. The lower the TIDE value, the better the ICB response. The above graphical analysis was performed using the R (v4.0.3) package ggplot2 (v3.3.3) and ggpubr (0.4.0). (**B**: cutoff at 50 years, i.e. Group 1 for CRC patients younger than 50 years and Group 2 for CRC patients older than 50 years; **C**: cutoff at 60 years for CRC patients; **D**: cutoff at 70 years for CRC patients.) **E**: The association between lymphocyte infiltration and good ICB response was explored using CRI iAtlas Portal in three different cohort studies using lymphocyte infiltration as a variable in melanoma samples [46,47] and glioblastoma samples (Prins 2019). **F**: In three different cohort studies, age was used as a variable to explore whether increasing age was associated with a better ICB response in melanoma and glioblastoma samples, which was analyzed by CRI iAtlas Portal.

CRC, Colorectal cancer; TIDE, Tumor Immune Dysfunction and Exclusion; ICB, Immune-checkpoint blockade; CD274: CD274 Molecule, Programmed Death Ligand 1, PD-L1; CTLA-4: Cytotoxic T-Lymphocyte Associated Protein 4; LAG3: Lymphocyte Activating 3; PDCD1LG2: Programmed Cell Death 1 Ligand 2, PD-L2; SIGLEC15: Sialic Acid Binding Ig Like Lectin 15; HAVCR2: Hepatitis A Virus Cellular Receptor 2, TIM-3; PDCD1: Programmed Cell Death 1, PD-1; TIGIT: T Cell Immunoreceptor With Ig And ITIM Domains.

Having previously found a negative correlation between age and CD8+ TILs in patients with CRC, we speculated whether age as an important clinical factor was also strongly associated with the response to ICB in these patients. Therefore, we further analyzed the relationship between age as an important clinical factor and the expression of immune checkpoints, as well as the prognosis and response to ICB therapy in patients with CRC. Through this analysis, we found that the expression levels of each immune checkpoint gene were significantly higher in patients with CRC older than 50 years than in those younger than 50 years (Figure 6a). The expression distribution of Cytotoxic T-Lymphocyte Associated Protein 4 (CTLA4), Lymphocyte Activating 3 (LAG3), Programmed Cell Death 1 Ligand 2(PDCD1LG2, PD-L2), and Sialic Acid Binding Ig Like Lectin 15 (SIGLEC15) had a significant statistical difference (p < 0.001).

To further estimate whether increasing age made a difference to ICB therapy response, we divided patients with CRC based on their ages as follows: 50 (Group 1 < 50 years, Group 2 > 50 years), at 60 (Group 1 < 60 years, Group 2 > 60 years), and at 70 (Group 1 < 70 years, Group 2 > 70 years), respectively, to see at which age exactly the patients responded better to ICB. We found that the older the patients, the better the response to ICB treatment, regardless of the age group. However, there was no statistically significant difference between a cutoff of 50 (Figure 6b) and a cutoff of 60 (Figure 6c). When we used 70 years as the cutoff, we found that patients with CRC responded significantly better to ICB when they were older than 70 years, and the difference was statistically significant (p = 0.019) (Figure 6d).

In addition, we found that in two cohort of Gide et al. [46] and Hugo et al. [47], skin cutaneous melanoma (SKCM) sample, the median number of lymphocyte infiltration was used as a cutoff to divide the two groups of melanoma samples into the upper half with high lymphocyte infiltration, and the lower half with low lymphocyte infiltration. We found that more lymphocyte infiltration meant better patient responses to ICB therapy. However, this result did not apply to the cohort of Prins et al., a cohort of glioblastoma samples (Figure 6e). Moreover, we found that the results of increase in age demonstrated a better ICB therapy response in Gide et al. [46], Hugo et al. [47] and Prins et al. [48], which means that not only in patients with CRC, but also in those with melanoma, patients with Glioblastoma (GBM) have not got similar results like melanoma patients (Figure 6g). Thus, ICB therapy may be a good choice for some specific tumors, such as CRC and melanoma, especially for older patients who cannot tolerate surgery.

### Discussion

Over the last few years, a substantial body of evidence has emerged showing the important function of infiltrating immune cells in tumor management and as a powerful prognostic factor in COAD and READ. TILs are useful in determining prognosis and evaluating immunotherapeutic effects, and TIL malfunction can lead to poor survival and early metastases [49]. As mentioned in the literature review, low levels of immune cell infiltration, as evaluated by T-cell and myeloid cell infiltration, are associated with poor outcomes in various solid tumors when compared to high immune infiltration [50,51]. What is more, a higher frequency of TILs, especially CD8+ TIL, is correlated with a better prognosis in CRC [52,53]. However, several reports have shown that Th2 cells and Treg T cells indicate a poor prognosis for CRC [54,55]. Therefore, which sub-group is the major component in TILs is of vital importance and directly related to the prognosis of CRC. One of the aims of this study was to determine the proportion or the infiltration level of TIL sub-groups in CRC and the CRC prognosis of each group.

The subsets of TILs are relatively conservative, even among different tumors in different patients. On the contrary, the relative proportions of each subgroup of TILs showed considerable heterogeneity, potentially suggesting that the balance between different immune cell states is a major factor in regulating the antitumor immune response. On the question of the TIL sub-groups infiltration level in CRC, the study found that the levels of infiltration from high to low were M2 macrophages, followed by resting CD4+ memory T cells, M0 macrophages, CD8 + T cells, activated CD4+ memory T cells, T cell follicular helper, and regulatory T cells in three TCGA cohorts. When we took the results of five different algorithms among five different TCGA tissue samples into comprehensive consideration, we found that the highest infiltrating proportion was still macrophages, followed by CD4 + T cells, mDC cells, CD8 + T cells, neutrophils, B cells, monocytes, and NK cells.

In order to eliminate the influence of tumor heterogeneity to the results, we used scRNA-seq database to further evaluate the proportion of TILs in four CRC GEO datasets. To our interest, macrophages were no longer the highest percentage; instead, conventional CD4 + T cells were. CD4+ TILs have received less attention as a prognostic biomarker in CRC, and their clinical importance in disease outcome is yet unknown. For the reason that CD4 + T cells contain different subsets, different subgroups of CD4 + T cells correspond to different outcomes of CRC. CD4+ CD45RO+ TILs represented good prognosis [56,57], while others were associated with poor outcomes. For instance, CD4+ CD25+ Regulatory T cells (Tregs) have immunosuppressive function. In many malignancies, the increase of Tregs within tumor tissues is thought to be a bad prognostic marker. As a vital marker of Tregs, Forkhead Box P3 (FOXP3) is commonly believed to reflect a poor prognosis [58–60]. However, other studies have shown that there are two different types of FOXP3 in CRC, inhibitory FOXP3 (FOXP3hi) and non-inhibitory FOXP3 (FOXP3lo), respectively, representing different prognoses and outcomes [61]. CRC with FOXP3lo TILs infiltration had a considerably better prognosis than CRC with FOXP3hi TIL infiltration [62]. Consistent with these findings, our study found that high level of CD8+ TIL or CD4+ TIL infiltration demonstrated better outcomes in CRC. The relationships among the infiltrating level of other subgroups and prognosis have been evaluated but have no significance of statistics (p > 0.05). This may be because of insufficient number of patients with CRC enrolled in the study.

Interestingly, we found that APC, TP53, TTN, PIK3CA, and KRAS were arranged from high to low according to the mutation frequency of CRC. In most instances, a larger percentage of TILs was found in the mutant status of a specific gene than in the wild type. The infiltrating level of subgroups of TILs from highest to lowest were always DC cells, CD8 + T cells, CD4 + T cells, neutrophils, B cells, and macrophages, regardless of whether the genes status was mutated or wild type or whether somatic copy number variation occurred or not. These results revealed that the proportion of TLIs of CRC is to a great extent relatively constant, which bear little relation to tumor mutated load and somatic copy number variation. However, even for same-species tumors, there is great heterogeneity existing in tumor microenvironment among different populations, which may generate the alteration of infiltrating level of TIL subgroups. In general, CD4+ TILs and CD8+ TILs account for higher proportion than other subsets of TILs, which is the reason why higher TIL infiltration demonstrated a better prognosis in most previously mentioned studies. Surprisingly, we found that CD8+ TILs and B plasma are negatively related to age. With advancing age, the infiltrating B plasma and CD8+ TILs reduce consequently. Less infiltrating B plasma and CD8+ TILs demonstrated poor prognosis of CRC. Our results provide a good explanation for why increasing age is a vital risk factor and related to poor prognosis of CRC.

A substantial body of evidence has emerged showing the important function of infiltrating immune cells in tumor control and as a powerful prognostic factor in CRC. In this study, the composition of CRC infiltrating immune cells was preliminarily analyzed by bioinformatics through public database, and the single-cell transcriptome database was innovatively used for further analysis. This provided new clues and bases for the correlation between TILs and CRCs. TILs participate in the occurrence and development of CRCs. Compared with the normal tissue, the composition of immune cells in CRC tissues has certain specificity, and infiltration features are not as specific gene mutation status or copy number variation change, which can provide a reference for the early screening and diagnosis of the disease. Patients of different ages may have different immune cell infiltration patterns, which can be used as a basis for clinical exploration of drug targets and targeted treatment to improve survival rate. CD4+ TILs and CD8+ TILs have certain application value in the prognostic prediction of CRC. This can be used as possible therapeutic indicators for new immunotherapy of CRC and screening a target population for possibly suitable adoptive cell therapy.

The conventional wisdom is that as age increases, TILs decrease, and the response to ICB therapy worsens. Our results demonstrate that CD8+ TILs decrease with age, yet ICB response is better particularly in patients with CRC older than 70 years, with a statistically significant difference in ICB response. This is associated to the heterogeneity of TILs, with a change in the proportion of infiltrated TILs and an upregulation of immune checkpoints with age, resulting in a better ICB response. In addition, competitive uptake of glucose in the tumor microenvironment is responsible for impaired T cell function, but the expression of amino acids, glutamine, fatty acids, and other metabolites or growth factors as well as the corresponding transporters on the cell surface are equally important factors affecting immune cell function and immunotherapy response. Furthermore, we noticed that the function and application value of glycans and glycosylation has gradually emerged in colon cancer, especially Programmed Death Ligand 1 (PD-L1, CD274) which is highly glycosylated, and the role of glycosylation in PD-L1 stability is regulated by the IL-6/Jak1 signaling, by affecting PD-L1 glycosylation. The root inhibits immune escape of tumor cells and enhances T cell killing [63], which is perhaps another reason for better response to ICB therapy.

However, there is still a major drawback. For instance, the study was established on the basis of only bioinformatics, which is not enough, and we have not explored the reasons for this result in-depth. The exact mechanism by which the predominant subpopulation of TILs affects TME and the ICB response is not known and will require further sufficient laboratory validation.

## Conclusion

Overall, the findings of the present study highlight the TIL infiltration proportion in CRC are constant to a great extent regardless of specific gene mutations or somatic copy number variation occurrence, which contribute to the early screening and diagnosis of CRC. In addition, the data corroborate that CD4+ TILs and CD8+ TILs have certain application values in the prognosis of CRC. The clinical features, for instance age, is negatively related to CD8+ TILs and B plasma infiltration. This research provides a timely and valuable study that increasing age, especially in patients with CRC older than 70 years, may have a better response to immunotherapy. Our study is valuable for immunotherapy and provides another effective option for many elderly patients who cannot tolerate surgical treatment.

## Supplementary Material

Supplemental Material

## Data Availability

The datasets analyzed for this study were obtained from the TIMER 2.0 (http://timer.cistrome.org), the TISCH (http://tisch.comp-genomics.org/home), and the GSE sc-seq samples (Zhang L, et al. Nature 2018; Wu TD, et al. Nature 2020; Zhang L, et al. Cell 2020), and the TCGA (https://portal.gdc.cancer.gov/).

## Highlights

● The infiltrating level of TIL subgroups in CRC has a constant proportion.

● CD4+ TILs and CD8+ TILs present a better prognosis for CRC.

● Age is negatively related to CD8+TILs, which may demonstrate a worse prognosis.

● Age may be a vital factor in immunotherapy of patients with CRC.

● Patients older than 70 years respond better to ICB.

## Abbreviations

CRC: Colorectal cancer; COAD: Colon cancer; READ: Rectum cancer; TILs: Tumor-infiltrating lymphocytes; scRNA-seq: Single-cell RNA-sequence; ACT: Adoptive cell therapies; TCGA: The Cancer Genome Atlas; GEO: Gene Expression Omnibus; GSE: Gene Expression Omnibus Series; TNM: Tumor Lymph Node Metastasis; TIMER: Tumor Immune Estimation Resource; TISCH: The Cancer Immune Single-Cell Hub; Treg: Regulatory T cells; OS: Overall survival; TP53: Tumor Protein P53; APC: APC Regulator Of WNT Signaling Pathway; PIK3CA: Phosphatidylinositol-4,5-Bisphosphate 3-Kinase Catalytic Subunit Alpha; KRAS: Oncogene KRAS2; SCNA: Somatic copy number variation; TIMER: Tumor Immune Estimation Resource; X-Cell: Cell types enrichment analysis; quanTIseq: QUANTISEQ R package; MCP-counter: MCP-counter R package; MDSC: Myeloid-derived suppressor cells; CR: Complete response; TIDE: Tumor Immune Dysfunction and Exclusion; TME: Tumor microenvironment; ICB: Immune-checkpoint blockade; SKCM: Skin cutaneous melanoma; GBM: Glioblastoma; FOXP3: Forkhead Box P3; TNN: Tenascin N; CTLA-4: Cytotoxic T-Lymphocyte Associated Protein 4; LAG3: Lymphocyte Activating 3; PD-L2/PDCD1LG2: Programmed Cell Death 1 Ligand 2; SIGLEC15: Sialic Acid Binding Ig Like Lectin 15; HAVCR2: Hepatitis A Virus Cellular Receptor 2, TIM-3; PDCD1: Programmed Cell Death 1, PD-1; TIGIT: T Cell Immunoreceptor With Ig And ITIM Domains.
