# Supplementary material for: Tumor-infiltrating lymphocyte: features and prognosis of lymphocytes infiltration on colorectal cancer
Source: Bioengineered. 2023 Jan 12;13(6):14872–88. doi: 10.1080/21655979.2022.2162660 (PMC9995135; doi:10.1080/21655979.2022.2162660)
Supplement: Supplemental Material [file KBIE_A_2162660_SM3843.zip › supplementary/Additonal file 6 Original Table 1 and Table 2.docx]

**Table 1 The multivariate Cox proportional hazard analyses of the subsets of CRC TILs.**

| **Clinical**  **characteristics/**  **subsets of TILs** | **TCGA-COAD**  **[N=253,n(live)=190, n(die)=63]** | | **TCGA-READ**  **[N=80, n(live)=66, n(die)=14]** | |
| --- | --- | --- | --- | --- |
|  | **HR（95%CI）** | **p-value** | **HR（95%CI）** | **p-value** |
| Age | 1.036(1.012-1.060) | **0.003**** | 1.135(1.031-1.25) | **0.010*** |
| Gender_male | 1.245(0.726-2.132) | 0.426 | 1.494(0.216-1.031) | 0.684 |
| Stage2 | 1.318(0.412-4.214) | 0.641 | 3.930(0.044-3.477) | 0.401 |
| Stage3 | 2.564(0.793-8.294) | 0.116 | 6.220(0.089-4.348) | 0.632 |
| Stage4 | 7.718(2.282-26.109) | **0.001**** | 1.970(0.018-2.187) | 0.186 |
| Race_Black | 0.541(0.064-4.569) | 0.573 | 2.148(0.000-Inf) | 0.998 |
| Race_White | 0.501(0.062-4.044) | 0.517 | 4.834(0.000-Inf) | 0.999 |
| Purity | 0.685(0.145-3.231) | 0.633 | 9.971(0.028-3.494) | 0.442 |
| B_cell | 32.559(0.042-25494.775) | 0.306 | 5.240(0.000-9.298) | 0.940 |
| CD8_T cell | 0.009(0.000-1.896) | 0.084 | 0.000(0.000-8.210) | **0.049*** |
| CD4_T cell | 0.079(0.000-24.608) | 0.386 | 0.000(0.000-4.523) | 0.457 |
| Macrophage | 39.692(0.110-14378.733) | 0.221 | 4.772(0.000-7.769) | 0.164 |
| Neutrophil | 0.073(0.000-3884.177) | 0.637 | 9.678(0.000-4.223) | 0.320 |
| Dendritic | 3.193(0.038-269.042) | 0.608 | 4.320(0.015-1.214) | 0.138 |

**Table 1:** Age and Stage4 were independent prognostic factors in COAD, similarly, age and CD8_T cell were independent prognostic factors in READ.

**Table 2 The correlation analysis between age and each subsets of TILs of CRC.**

| **Tissue Type**  **Z-score** | **CD8+** | **CD4+** | **CD4+**  **naive** | **CD4+**  **mem** | **B**  **mem** | **B**  **plasma** | **Mono** | **Macro** | **NK** |
| --- | --- | --- | --- | --- | --- | --- | --- | --- | --- |
| TCGA-COAD  (N=458) | n.s | 2.432 | 2.904 | n.s | 2.649 | n.s | n.s | n.s | 2.122 |
| TCGA-READ  (N=166) | **-2.212*** | n.s | n.s | 2.5 | n.s | **-3.133*** | 2.157 | 2.651 | n.s |

******Z-score：increased risk (Z>0, P<0.05)

Z-score：decreased risk (Z<0, P<0.05)

n.s：not significant (P>0.05)

**Table 2:** We found that CD8+TILs and B plasma are negatively related to age. With age changing, the infiltration features of subgroups of TILs also change consequently. CD4+mem:CD4+ memory; B mem: B memory; Mono: Monocyte; Macro: Macrophage.
