# Supplementary material for: Tumor-infiltrating lymphocyte: features and prognosis of lymphocytes infiltration on colorectal cancer
Source: Bioengineered. 2023 Jan 12;13(6):14872–88. doi: 10.1080/21655979.2022.2162660 (PMC9995135; doi:10.1080/21655979.2022.2162660)
Supplement: Supplemental Material [file KBIE_A_2162660_SM3843.zip › supplementary/Additonal file 6_Figures.pptx]

## Slide 1
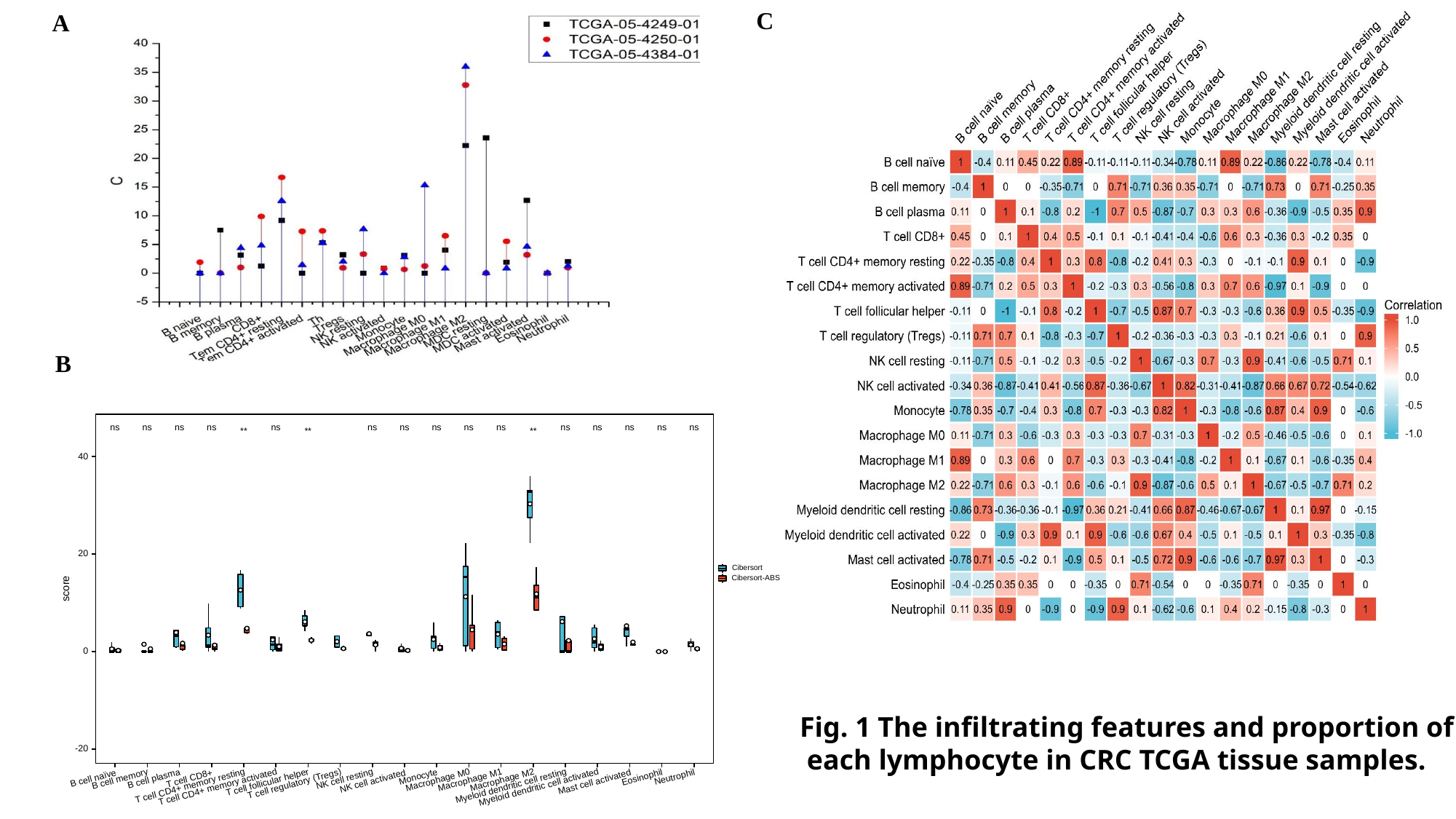

C
A
B
ns
ns
ns
ns
ns
ns
ns
ns
ns
ns
ns
ns
ns
ns
ns
**
**
**
 40
 20
Cibersort
Cibersort-ABS
score
 0
-20
Monocyte
Neutrophil
Eosinophil
B cell plasma
B cell memory
NK cell resting
B cell naïve
T cell CD8+
Macrophage M0
Macrophage M1
Macrophage M2
NK cell activated
T cell follicular helper
Mast cell activated
T cell regulatory (Tregs)
T cell CD4+ memory resting
Myeloid dendritic cell resting
T cell CD4+ memory activated
Myeloid dendritic cell activated
Fig. 1 The infiltrating features and proportion of
 each lymphocyte in CRC TCGA tissue samples.

## Slide 2
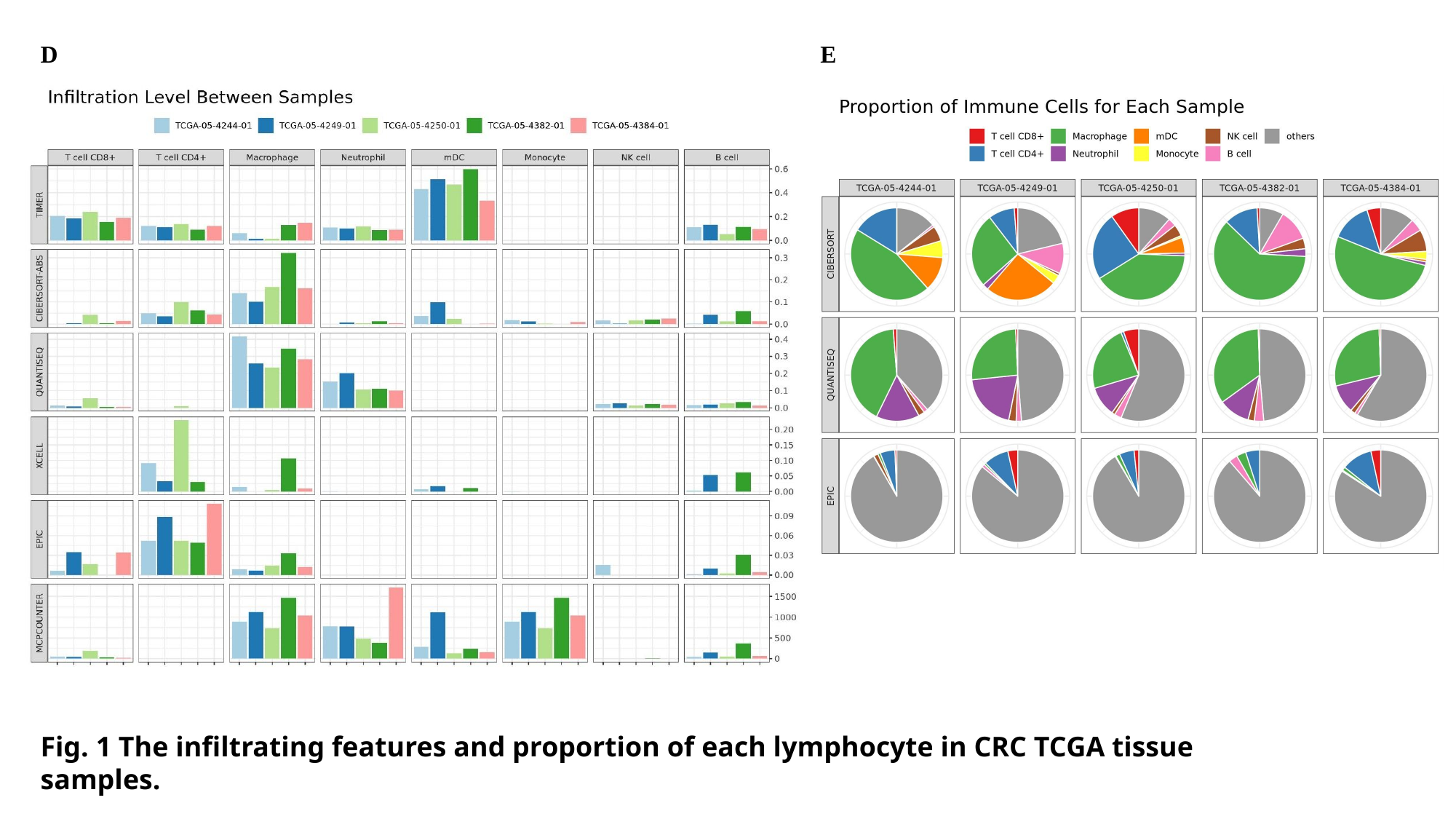

D
E
Fig. 1 The infiltrating features and proportion of each lymphocyte in CRC TCGA tissue samples.

## Slide 3
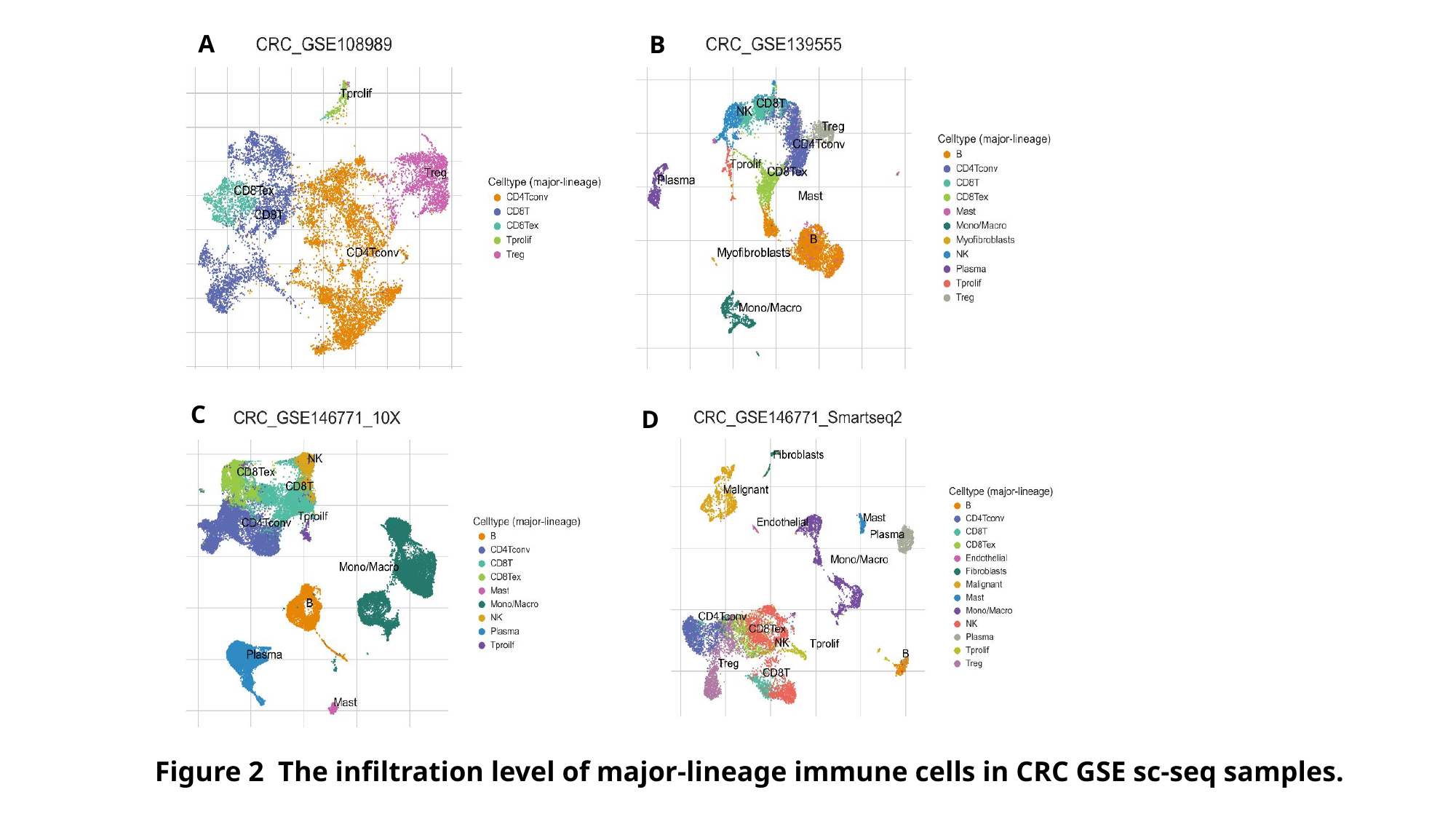

A
B
C
D
 Figure 2 The infiltration level of major-lineage immune cells in CRC GSE sc-seq samples.

## Slide 4
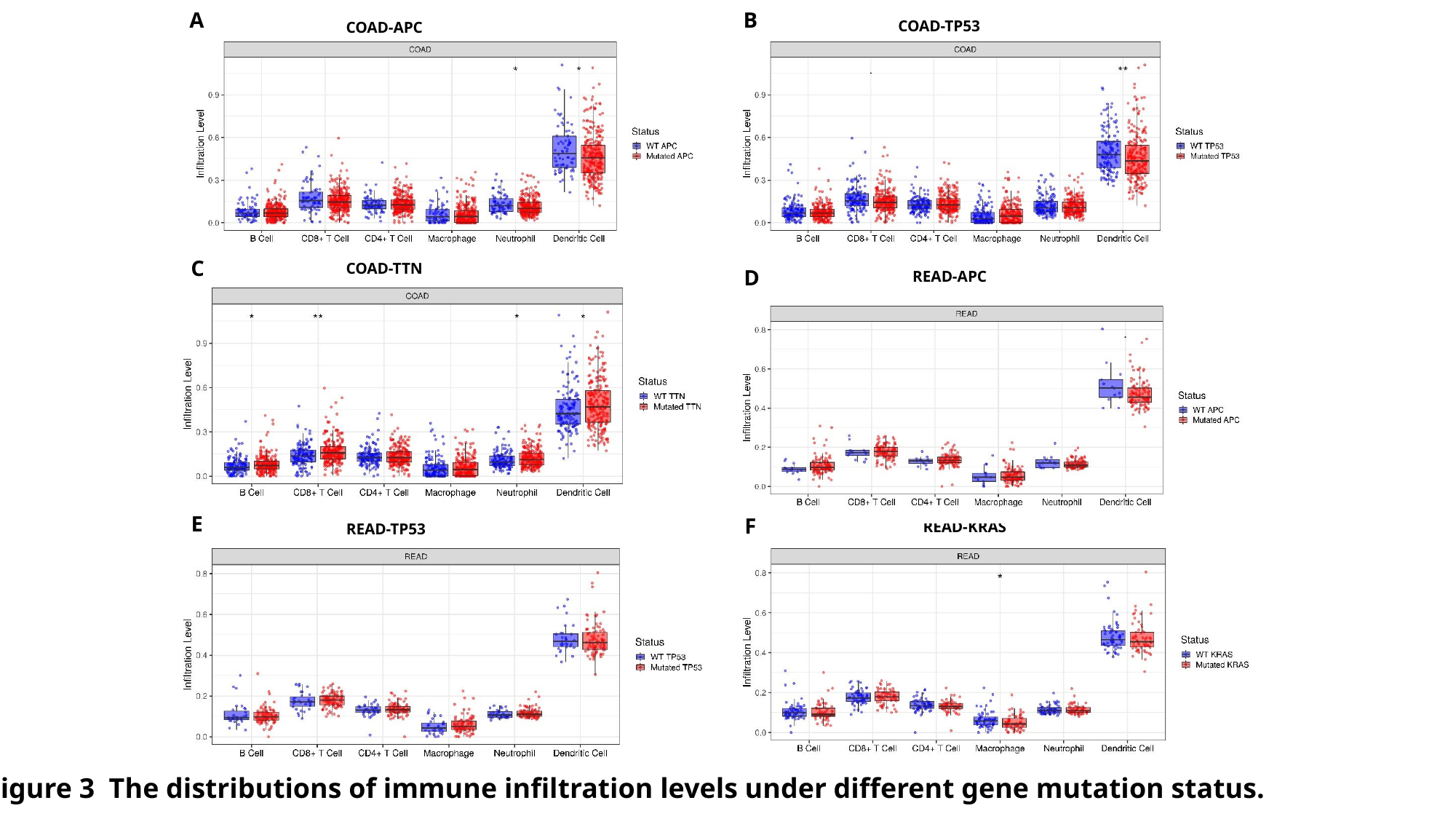

B
A
COAD-TP53
COAD-APC
C
COAD-TTN
D
READ-APC
READ-APC
E
F
READ-KRAS
READ-TP53
Figure 3 The distributions of immune infiltration levels under different gene mutation status.

## Slide 5
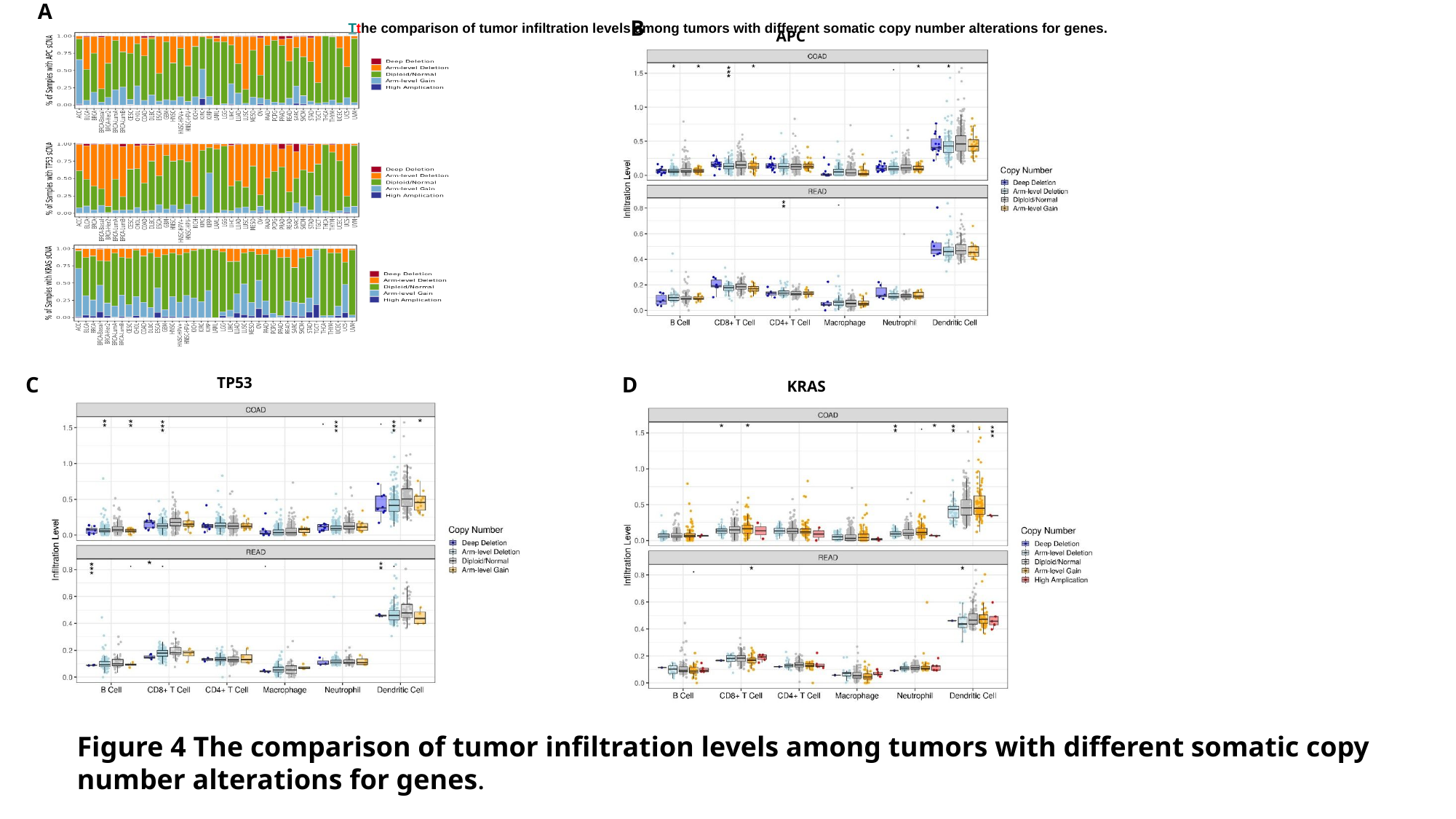

Tthe comparison of tumor infiltration levels among tumors with different somatic copy number alterations for genes.
A
B
 APC
C
D
 TP53
 KRAS
 TP53
Figure 4 The comparison of tumor infiltration levels among tumors with different somatic copy number alterations for genes.

## Slide 6
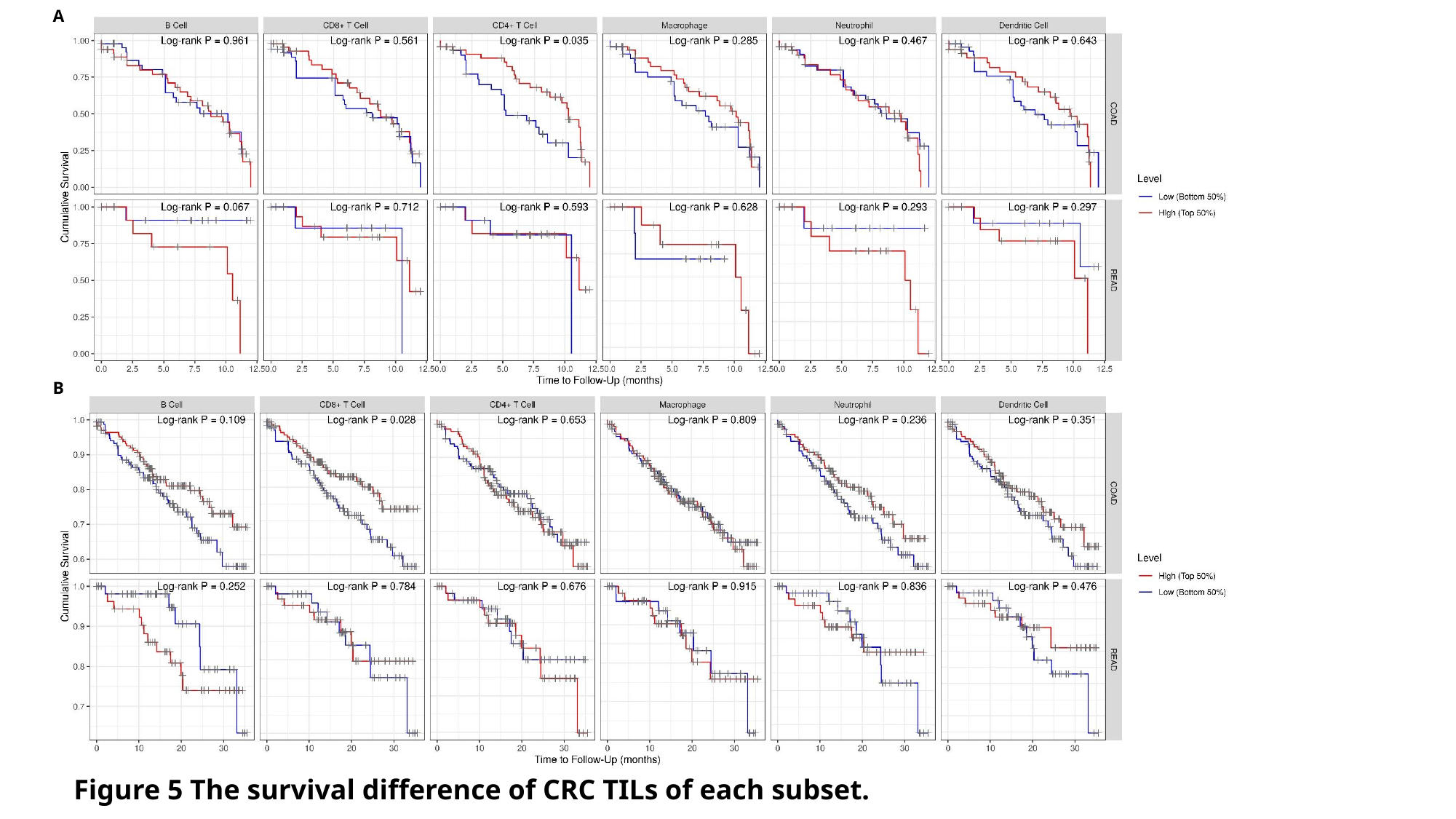

A
B
Figure 5 The survival difference of CRC TILs of each subset.

## Slide 7
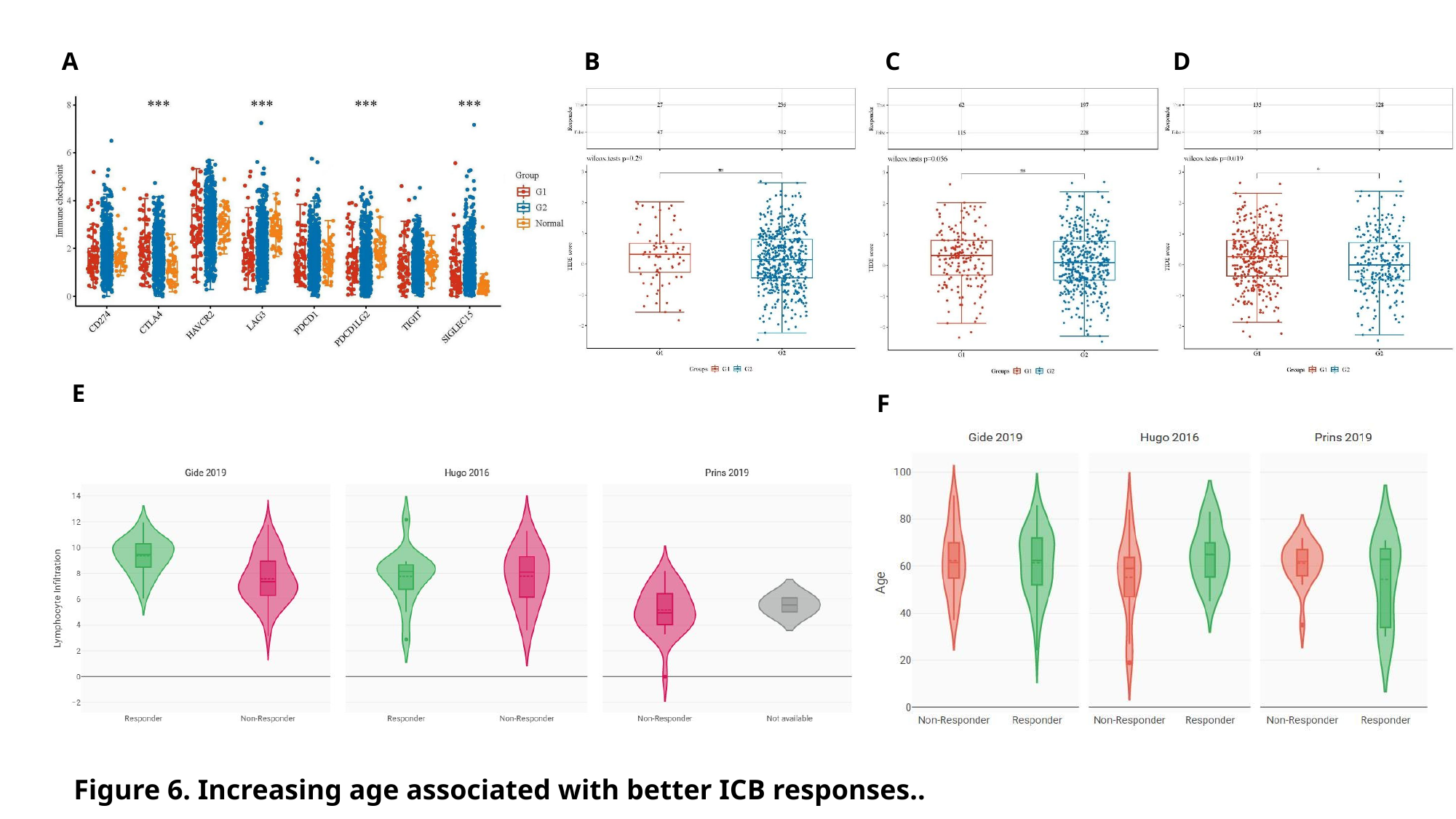

A
B
C
D
E
F
Figure 6. Increasing age associated with better ICB responses..

## Slide 8
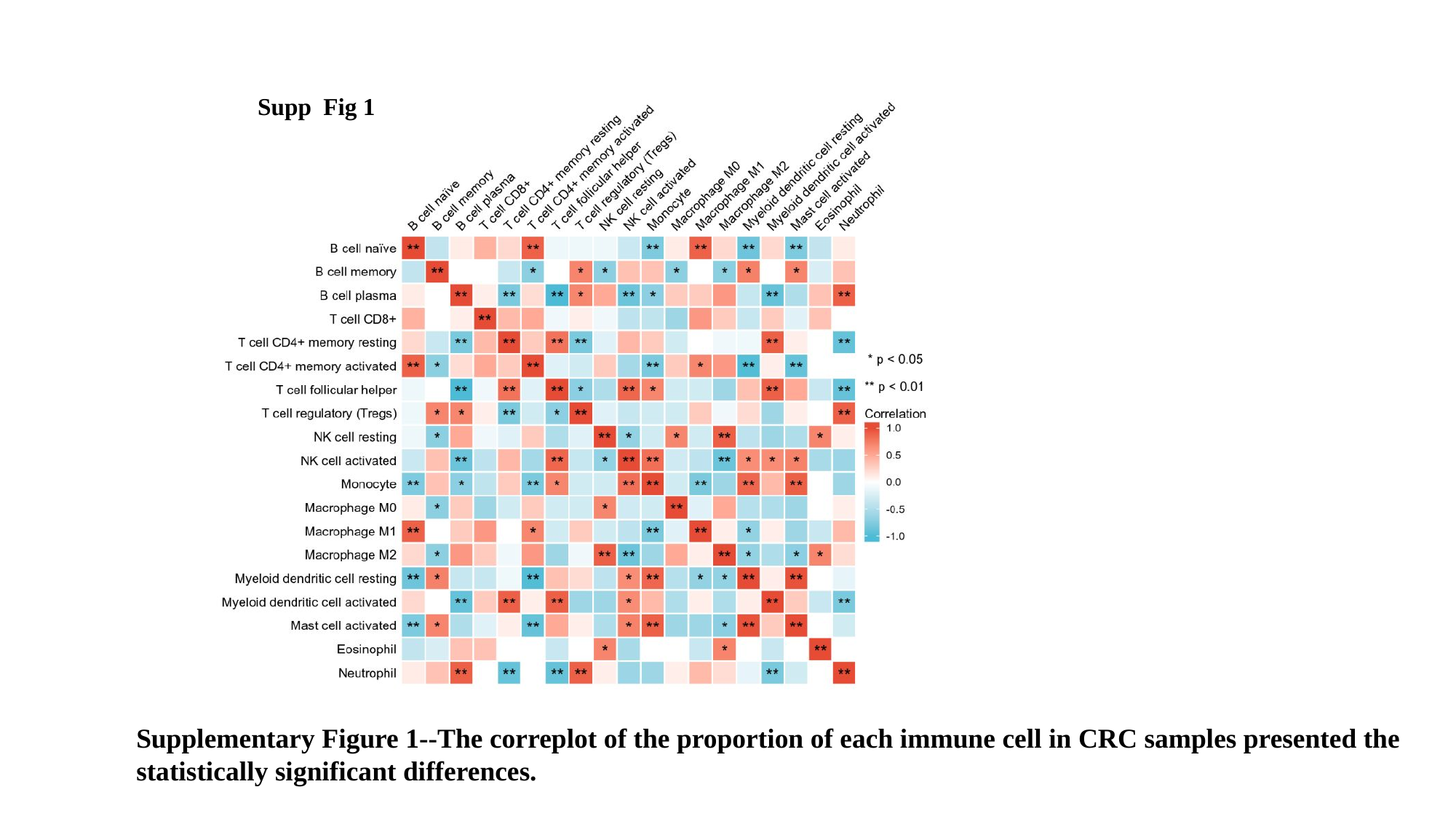

Supp Fig 1
Supplementary Figure 1--The correplot of the proportion of each immune cell in CRC samples presented the statistically significant differences.
